# Introduction to the Toxins Special Issue on Botulinum Neurotoxins in the Nervous System: Future Challenges for Novel Indications

**DOI:** 10.3390/toxins12090601

**Published:** 2020-09-17

**Authors:** Siro Luvisetto

**Affiliations:** National Research Council of Italy-CNR, Institute of Biochemistry and Cell Biology (IBBC), via Ercole Ramarini 32, Monterotondo Scalo, 00015 Roma, Italy; siro.luvisetto@cnr.it

*Botulinum* toxins (BoNTs) are a true wonder of nature. Like Dr. Jekyll and Mr. Hyde, they have a double “personality”, making them unique among the toxins of bacterial origin. As Dr. Jekyll, BoNTs are drugs approved for a variety of clinical conditions while, as Mr. Hyde, they are one of the most dangerous toxins, causing botulism. In the past, many studies have extensively investigated the mechanism of action of BoNTs, showing a variety of apparently different mechanisms which have in common the block of the cholinergic transmission, mainly at the neuromuscular junction. These discoveries gave an extraordinary consensus to therapeutical use of BoNTs in human pathologies characterized by excessive muscle contractions, i.e., hypercholinergic dysfunctions including torticollis, blepharospasms, dystonia, and so on. Recently, the list of human disorders in which treatments with BoNTs have produced, or are expected to produce, beneficial effects is long and continuously growing. The ambitious goal of this Special Issue of *Toxins* was to provide an up-to-date picture on the state of studies for the development of new therapeutic treatments with BoNTs, mainly with serotypes A (BoNT/A) or B (BoNT/B). This Editorial is an introduction to the 25 contributions (14 research and 11 review papers) collected in this Special Issue of *Toxins*, which I strongly invite you to read in their original versions.

The first three papers focus on the treatment with BoNT/A of limb essential tremors (ETs), a neurology condition characterized by persistent postural, or kinetic, tremor due to involuntary rhythmic muscle activity of the upper or lower limbs, neck, and trunk. In detail, Zakin and Simpson [1] contributed with an overview on the techniques for BoNT/A injection, together with muscle targeting techniques, in the treatment of ETs. Niemann and Jankovic [2] reported the results of a retrospective study performed on a large database of patients treated with BoNT/A for hand tremor of different origins, mainly ETs but also dystonic, Parkinsonian, and cerebellar. Finally, Samotu et al. [3] analyzed the efficacy of BoNT/A in one open-label trial, with participants affected by Parkinson’s disease (PD) and ETs.

Three clinical studies investigated the effects of BoNT/A in post-stroke spasticity (PSS), a common impairment arising from involuntary activation of muscles that often appears after stroke. Rosales et al. [4] performed a clinical trial (ONTIME) in post-stroke patients with spastic paresis, and analyzed the impact of BoNT/A on symptomatic spasticity progression. ONTIME provided evidence that an early BoNT/A injection improved muscle tone, delayed time to appearance of PSS symptoms, and significantly increased time until re-injection. Shin et al. [5] reported results from an open-label pilot study demonstrating that BoNT/A injection into finger and wrist flexors, followed by electrical stimulation of the finger extensor, improved active hand function in chronic stroke patients. Ianieri et al. [6] recalled the importance of performing an accurate evaluation of spasticity to determine how invalidating the symptoms are in order to personalize, for each patient, the optimal doses of BoNT/A, muscles, and injection time.

Another series of papers focuses on new dermatological uses of BoNTs. Kim et al. [7] contributed with a review for the use of BoNT/A in several off-label dermatological indications, including regenerative treatments of hypertrophic scarring and keloids, postoperative scar prevention, rosacea and facial flushing, and post-herpetic neuralgia, all conditions associated with hyperhidrosis, oily skin, psoriasis, and itching. Itching constitutes another dermatological condition where application of BoNT/A may exert beneficial effects, especially in the treatment of neuropathic itching, a debilitating symptom appearing secondary to several skin, systemic, metabolic, and psychiatric disorders. The action of BoNT/A as an antipruritic agent is exhaustively reviewed by Gazerani [8] who summarizes all the evidence in favor both in animal models and in healthy human volunteers, and in many clinical conditions. The mechanism originating the antipruritic effects of BoNTs is discussed by Gazerani and also by Ramachandran et al. [9], who also give a possible explanation. The authors, analyzing the antipruritic effects of both BoNT/A and B in murine models, showed that both BoNT/A and B exert antipruritic effects in a mast cell/histamine-dependent and -independent manner.

Pain is another condition where the use of BoNTs is very promising. Different approaches have been adopted to treat chronic pain and, among others, the use of BOTOX^®^ (commercial preparation of BoNT/A from Allergan, Inc., Irvine, CA, USA) has been recently authorized as a novel pharmacological indication for the prophylaxis of chronic migraine. In this context, Ion et al. [10] reported the results from a prospective study on the effect of a new BoNT/A formulation, namely XEOMIN^®^ (commercial preparation of BoNT/A from Merz Pharmaceuticals, Inc., Frankfurt am Main, Germany), injected in patients with refractory chronic migraine. Unlike the other commercial preparationd of BoNT/A, XEOMIN^®^ benefits from the absence of binding albumin protein, minimizing allergic reactions. The authors proved that XEOMIN^®^ can be a prophylactic treatment in chronic migraine, effectively reducing the number of attack days, the number of migraine episodes per day, and the drug intake. Expanding the therapeutic uses of BoNTs, not only in pain, but also for overactive bladder, neurogenic detrusor overactivity, osteoarthritis, and wound healing, is exhaustively reviewed by Fonfria et al. [11]. The authors not only reviewed the effects of BoNTs, remote from injection sites, but also the effects of novel formulations, including modified and recombinant toxins, and of novel delivery methods, including transdermal, transurothelial, and transepithelial methods. Another potential analgesic effect of BoNTs is reviewed by Sandahl Michelsen et al. [12], who analyzed the results from a series of studies examining the effect of BoNT/A in alleviating pain in children with cerebral palsy (CP). The authors emphasize the difficulty concerning the treatment of pain in children with cerebral palsy (CP), a physical disability that affects the development of movement and posture in children and a neurological disorder in childhood caused by damage to either the fetal or the infant brain.

Continuing on the topic dedicated to the pain, Lee et al. [13] tested the efficacy of a lumbar sympathetic block with BoNT/A and B as pain therapy for complex regional pain syndrome (CRPS), a neuropathic pain syndrome causing spontaneous pain and allodynia. They found that the lumbar sympathetic block, with both BoNTs serotypes, constitutes a safe method to treat CRPS and, more surprisingly, BoNT/B is more effective and longer lasting than BoNT/A. The effects of BoNTs on CRPS as well as other neuropathic pain are also reviewed by Park et al. [14]. In addition to CRPS, this review also reports a complete overview of clinical studies of BoNT effects for central neuropathic pain, such as neuropathic pain after spinal cord injury, post-stroke shoulder pain, and central pain associated with multiple sclerosis. 

In a basic science study, Finocchiaro et al. [15] compared the effect of BoNT/A and B in counteracting neuropathic pain in a murine model of sciatic nerve injury. The results confirmed that BoNT/A reduces neuropathic pain over a long period of time and, in parallel, it induces an acceleration of the regenerative processes of injured nerves, improving the functional recovery of the injured limb. BoNT/B can also reduce neuropathic pain over a long period of time, but, compared to BoNT/A, this reduction is not accompanied by an improvement in functional recovery. Finally, in an interesting review, Rojewska et al. [16] discussed whether and how BoNT/A reduces the development of neuropathic pain, with particular emphasis on spinal neuron–glia interactions and on the role of glial cells in BoNT/A-induced analgesia. 

Another experimental study presented by Wang et al. [17] reported the nerve regeneration effects of BoNT/A on injured spinal cords in rats. What renders this paper unusual is the use of the BoNT/A heavy chain (BoNT/A-HC) as a catalytic subunit. This is completely in disagreement with the canonical view that BoNT/A-HC is the subunit necessary to bind to the vesicle presynaptic membrane and to translocate, inside the vesicle, the BoNT/A light chain (BoNT/A-LC), which constitutes the catalytic subunit which, by cleaving the SNARE proteins, blocks the neuronal transmission. The authors found that local application of BoNT/A-HC to the site of spinal cord injury significantly induced an increased expression of growth-associated protein, together with a stimulation of neurite outgrowths. The mechanism by which BoNT/A-HC favors the relief of spinal motor dysfunction after nervous injury remains unknown.

As for novel indications of BoNTs, we should not forget that BoNTs have been considered as agents for inducing controlled paralysis in different muscles of the oral, maxillofacial, and temporomandibular joint region, with the aim to treat dysfunction and dislocation in clinical orthodontics and maxillofacial surgery. Clinical applications of BoNTs in treatment for the correction of severe malocclusion-associated problems, including occlusion after orthognathic surgery and mandible fracture, are reviewed by Seok and Kim [18]. This particular application of BoNTs is based on the principle that the induction of controlled paralysis of masticatory muscles reduces the tensional force to the mandible and prevents relapse, and affects maxillofacial bone growth and dental occlusion. Yoshida [19] performed a study comparing the treatment outcome after intramuscular injection of BoNTs in patients with recurrent temporomandibular joint dislocation (TMD). Restivo et al. [20] reported an interesting effect of BoNT/A in reducing hypersalivation in patients with neurological diseases of different etiologies, including Parkinson’s, amyotrophic lateral sclerosis, brain injury, and cerebral palsy. 

Two reviews focus on new therapeutic approaches of BoNTs in gynecology and urinary tract dysfunctions. In the first, Moga et al. [21] made a literature review regarding the efficiency of BoNT/A in the treatment of chronic pelvic pain, vaginismus, vulvodynia, and overactive bladder or urinary incontinence. In the second, Jhang and Kuo [22] did a literature review regarding treatment of neurogenic lower urinary tract dysfunction, such as overactive bladder, neurogenic detrusor overactivity, interstitial cystitis, urethral sphincter dyssynergia, dysfunctional voiding, benign prostate hyperplasia, and chronic prostatitis.

Moving on to completely different topics, Caleo and Restani [23] contributed with a review describing the experimental use of BoNTs as a tool to block synaptic function in specific brain areas, with central delivery of BoNTs used to treat pathological brain conditions such as epilepsy, cerebral ischemia, Parkinson’s, and prion disease. For obvious reasons, primarily toxicity and toxin diffusion, these studies are still limited to animals. An example of this unusual utilization of BoNT/A is also reported by Antipova et al. [24], who injected toxin directly into the striatum of mice and compared the motor behavior. The authors speculate that locally applied BoNTs could be useful for treating brain dysfunctions that require the deactivation of local brain circuitry. 

The last paper was contributed by Bano et al. [25]. This paper reports a relevant observation on the fact that a tetanus neurotoxin (TeNT), a relative of BoNT/B produced by a *Clostridia tetani* strain, is neutralized by antisera raised against BoNT/B. This finding implicates that, although TeNT is not considered a food-borne pathogen, it can be present in foodstuffs and interfere with the detection of *Clostridia botulinum* by the mouse test, giving rise to misleading results. It is interesting to recall that humans are not usually vaccinated against type B botulism but citizens in many countries are regularly vaccinated for tetanus. It might be interesting to investigate whether the human vaccine for type B botulism also protects from certain isoforms of TeNTs.

In conclusion, the papers included in this Special Issue of *Toxins* contributed to the advancement of the state of the art in the novel therapeutic uses of BoNTs. Furthermore, many of the published studies focused on emerging or less investigated applications of BoNTs, in particular for pathologies, thus providing the scientific community with new data supporting better knowledge of the contributions given by BoNTs to improve the health of humanity.
